# Weighted Gene Co-Expression Network Analysis Reveals Key Genes and Potential Drugs in Abdominal Aortic Aneurysm

**DOI:** 10.3390/biomedicines9050546

**Published:** 2021-05-13

**Authors:** Ke-Jia Kan, Feng Guo, Lei Zhu, Prama Pallavi, Martin Sigl, Michael Keese

**Affiliations:** 1 Department of Surgery, Medical Faculty Mannheim, Heidelberg University, 68167 Mannheim, Germany; Kejia.Kan@medma.uni-heidelberg.de (K.-J.K.); Feng.Guo@medma.uni-heidelberg.de (F.G.); Lei.Zhu@medma.uni-heidelberg.de (L.Z.); Prama.Pallavi@medma.uni-heidelberg.de (P.P.); 2 European Center of Angioscience (ECAS), Medical Faculty Mannheim, Heidelberg University, 68167 Mannheim, Germany; 3 German Cancer Research Center (DKFZ), Junior Clinical Cooperation Unit Translational Surgical Oncology (A430), 69120 Heidelberg, Germany; 4 First Department of Medicine, Medical Faculty Mannheim, Heidelberg University, 68167 Mannheim, Germany; martin.sigl@umm.de

**Keywords:** abdominal aortic aneurysm, weighted gene co-expression network, key module, hub gene, functional enrichment, drug–gene prediction

## Abstract

Abdominal aortic aneurysm (AAA) is a prevalent aortic disease that causes high mortality due to asymptomatic gradual expansion and sudden rupture. The underlying molecular mechanisms and effective pharmaceutical therapy for preventing AAA progression have not been fully identified. In this study, we identified the key modules and hub genes involved in AAA growth from the GSE17901 dataset in the Gene Expression Omnibus (GEO) database through the weighted gene co-expression network analysis (WGCNA). Key genes were further selected and validated in the mouse dataset (GSE12591) and human datasets (GSE7084, GSE47472, and GSE57691). Finally, we predicted drug candidates targeting key genes using the Drug–Gene Interaction database. Overall, we identified key modules enriched in the mitotic cell cycle, GTPase activity, and several metabolic processes. Seven key genes (CCR5, ADCY5, ADCY3, ACACB, LPIN1, ACSL1, UCP3) related to AAA progression were identified. A total of 35 drugs/compounds targeting the key genes were predicted, which may have the potential to prevent AAA progression.

## 1. Introduction

Abdominal aortic aneurysm (AAA) is a localized dilation or bulging of the abdominal aorta, commonly occurring in the infrarenal region [1]. Most patients with AAA remain asymptomatic for years or even decades. It is estimated that around 200,000 AAA rupture cases are diagnosed worldwide annually, and the mortality after rupture remains around 80% [2,3,4].

Currently, AAA requiring intervention, e.g., large aneurysms with a diameter more than 5.5 cm, aneurysms that expand rapidly in a short period, or aneurysms that compromise the perfusion to distant organs are indicated for open surgical or endovascular aortic repair. However, the outcomes from these measures are not so satisfactory [5,6]. For patients with small AAAs or those who are not eligible for AAA repair, close aneurysm surveillance and adjuvant therapy are recommended [5]. So far, no effective pharmacological treatments have been developed to prevent AAA growth or rupture [7,8]. Hence, there is a need to elucidate the possible mechanisms of AAA progression and explore corresponding pharmaceutical treatments.

A number of preclinical mouse AAA models have been developed to understand the pathogenesis of AAA [9,10]. Among these models, angiotensin II-infused ApoE^−/−^ mice are the commonly used [11,12,13,14,15]. Although the inherent pathology of aneurysm is different between mice and humans, it shares some of the important properties of human AAA, like pronounced inflammatory responses and aortic rupture [11,12,13,14,15]. Based on the findings from mouse models and human samples, AAA is currently accepted as an inflammation-driven disease, as many related processes (such as infiltration of macrophages, neutrophils, B cells and T cells, and activation of inflammatory pathways) were found both in humans and mice [16,17,18,19]. Overactivation of the inflammatory response leads to the destruction of aortic media through the release of proteolytic enzymes and the death of vascular smooth muscle cells, which further promote AAA development [20].

Several studies based on the high-throughput microarray profiling further confirmed the involvement of the above biological processes in AAA, including the immune response, chronic inflammation, and reactive oxygen species [21,22,23]. Dozens of genes related to AAA development were identified through gene expression profiles [24,25,26]. However, these studies exclusively focused on the differentially expressed genes (DEGs) between AAA and control groups, which ignored some key genes that are highly correlated to specific sample traits of AAA. Weighted gene co-expression network analysis (WGCNA) is a bioinformatics algorithm developed by Horvath et al. [27]. By constructing a scale-free weighted network, WGCNA can investigate biologically meaningful gene sets connected to sample features and explore inner module hub genes that are highly associated inside the co-expression module. WGCNA has been successfully used to identify key modules and hub genes related to cardiovascular diseases, such as atherosclerosis, heart failure, and acute myocardial infarction [28,29,30]. So far, data collected at different time points of AAA progression have not been subjected to WGCNA analysis to identify the critical modules and hub genes.

In this study, WGCNA analysis was performed using the explore dataset GSE17901 in the Gene Expression Omnibus (GEO) database. Key modules of AAA development and hub genes in each module were identified. Gene functional enrichment analysis of key modules was applied to show their potential biological activities. Hub genes were screened in the STRING database and further selected in the Cytoscape software (San Diego, CA, USA). Key genes from hub genes were validated using mouse AAA model GSE12591 dataset and human AAA sample GSE7084, GSE47472, and GSE57691 datasets. Candidate drugs for AAA treatment were screened in the Drug Gene Interaction Database (DGIdb) based on the above-identified key genes.

## 2. Materials and Methods

### 2.1. Data Sources and Preprocessing

**Figure 1:**
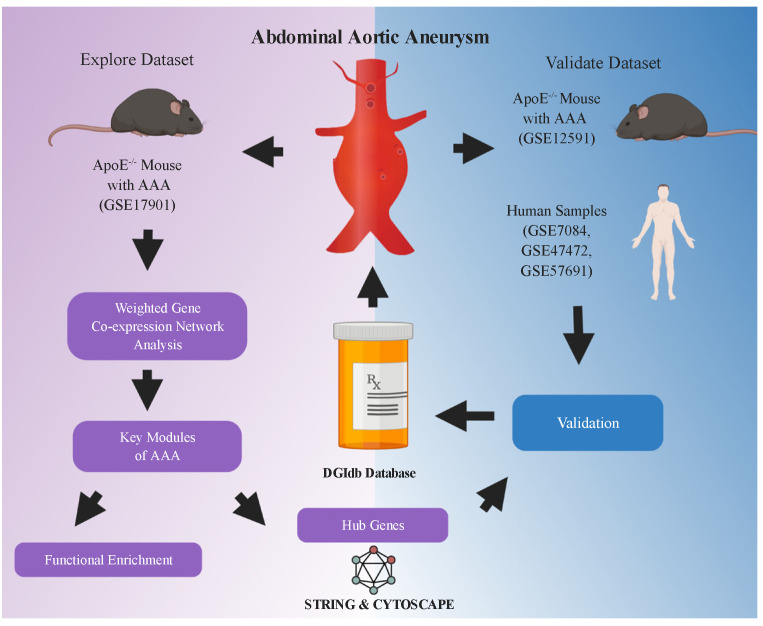
Flowchart of analysis in the study. GSE17901 was a mouse dataset containing AAA samples collected on day 7, day 14 and day 28, which was used for exploring the key modules and hub genes related to AAA progression. Hub genes were identified through the STRING database and Cytoscape software (San Diego, CA, USA). Key genes were further selected from the hub genes and validated in the mouse (GSE12591) and human (GSE7084, GSE47472 and GSE57691) AAA datasets. Finally, potential drugs or compounds targeting these key genes were screened in the DGIdb database. AAA: abdominal aortic aneurysm. The flowchart was created with BioRender.com (accessed on 11 April 2021).

The workflow of this study is shown in Figure 1. Datasets related to AAA—GSE17901, GSE12591, GSE7084, GSE47472 and GSE57691 (Table 1) were downloaded from the GEO database (accessed on 1 April 2020 from https://www.ncbi.nlm.nih.gov/geo/). In the explore dataset GSE17901 [26], aortic samples were taken on day 7, day 14, and day 28 from ApoE^−/−^ mice treated by angiotensin II or saline. The diameters of the treated aortas increased throughout the 28-day course, which we defined as the progression of AAA, so samples with AAA (*n* = 18) were selected for weighted gene co-expression network (WGCNA) analysis. Mouse dataset (GSE12591) and human datasets (GSE7084, GSE47472, and GSE57691) were used to validate the hub genes. The GSE12591 dataset included 18 mouse aortas exposed to saline (*n* = 6) or angiotensin II (*n* = 12) infusion [25]. The GSE7084 included control samples (*n* = 10) and AAA samples from patients (*n* = 9) [24]. The GSE47472 contained AAA neck specimen (*n* = 14) and normal aortic tissue from organ donors (*n* = 8). The GSE57691 included AAA samples (*n* = 49) and normal aortic specimens of organ donors (*n* = 10) [31]. Each dataset was processed by background correction, including removal of batch effect using the sva R package (version 3.12) and quantile normalization with the limma R package (version 3.38.3) [32] for further analysis.

### 2.2. Construction of WGCNA

The WGCNA R package (version 1.69) was used to perform the weighted co-expression network analysis. Genes with the top 25% variance from the explore dataset GSE17901 were selected for the following analysis step. Using the pick Soft Threshold function, the soft-thresholding power was determined and used to construct a scale-free network. Thereafter, gene co-expression modules were identified using the one-step network construction method and labeled with different colors. The reassign threshold was set at 0.25, and the minimum number of genes in each module was 30.

### 2.3. Selection of Key Modules Corresponding to Sample Traits

To explore the key modules that are significantly associated with sample traits of AAA, we calculated the relevancy between module eigengene (ME), which summarizes each module’s expression profiles. The correlation results were shown using the ggcorrplot R package (version 0.1.3) [33]. Furthermore, Gene Significance (GS) was quantified by the absolute value of the association between the gene expression and sample trait. In every module, measurement of module membership (MM) was defined as the correlation of the ME and gene expression profile. Modules with high significance (*p*-value < 0.05) and relationships (correlation >0.6 or <−0.6) were defined as key modules of AAA and used for hub gene selection.

### 2.4. Functional Enrichment Analysis of the Key Modules

To understand the biological activities of genes in key modules, we conducted Gene Ontology (GO) function enrichment analysis and Kyoto Encyclopedia of Genes and Genomes (KEGG) pathway analysis with the clusterProfiler R package (version 3.10) [34]. Adjusted *p*-value < 0.05 was considered a statistically significant difference in enrichment analysis, and the top 10 of each analysis were extracted for visualization.

### 2.5. Identification of Hub Genes in the Key Modules

Hub genes are those that have a high degree of intramodular connectivity. In this study, hub genes were defined as the top 10% of genes from key modules with the highest connectivity. We uploaded them into the search tool for the retrieval of the interacting genes (STRING) website (accessed on 1 May 2020 from www.string-db.org) for protein–protein interaction analysis, choosing the confidence >0.4 [35]. Cytoscape software (San Diego, CA, USA) was used for network visualization and hub gene selection [36]. The top 10 hub genes in each module were selected with the maximal clique centrality (MCC) method using cytoHubba plugin software in Cytoscape (San Diego, CA, USA) [37].

### 2.6. Hub Genes Validation and Key Genes Selection

The validation of hub genes was performed by comparing the normalized gene expression value between control and AAA groups. The validated datasets GSE12591, GSE7084, GSE47472, and GSE57691 were downloaded from the GEO database, and data were preprocessed as mentioned before. In the GSE12591 mouse dataset, the gene expression of the selected hub genes in AAA and controls were compared, and genes with *p* < 0.05 were confirmed as the key genes. In the GSE7084, GSE47472, and GSE57691 human datasets, genes were extracted as described for dataset GSE12591. Genes with *p* < 0.05 were confirmed as the key genes. Common genes in both the mouse dataset and human datasets were defined as the final key genes.

### 2.7. Predication of Drug–Gene Interaction

The Drug–Gene Interaction Database (DGIdb) (accessed on 8 June 2020 from http://www.dgidb.org/) is an online database of drug–gene interaction data aggregated from various sources, including several drug databases (DrugBank, PharmGKB, ChEMBL), clinical trial databases, and literature from PubMed [38]. The selected key genes that were considered the potential pharmaceutical targets for AAA treatment were imported into DGIdb to explore existing drugs or small organic compounds. Results were displayed using the R packages ggplot2 (version 3.2.1) [39] and ggalluvial (version 0.11.1) [40].

### 2.8. Statistical Analysis

To define the statistical significance of differences between the two groups, we performed analysis using a non-parametric test or t-test based on data distribution characteristics. All analyses were conducted with R software (version 3.5.5). *p*-value < 0.05 was assigned statistical significance.

## 3. Results

### 3.1. Construction of Weighted Gene Co-Expression Network

**Figure 2:**
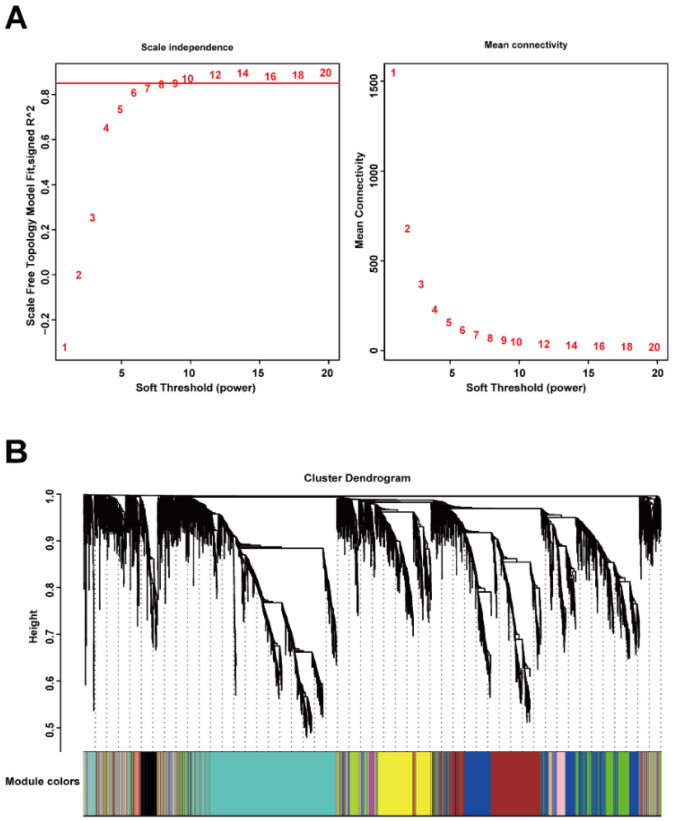
Construction of gene co-expression network by WGCNA. (**A**) Determination of soft-thresholding power for scale-free network construction. Here, we set the coefficient threshold at 0.85, and the soft-threshold was 9; (**B**) cluster analysis of the dendrogram and identification of co-expressed modules. In this study, we got 15 related co-expression modules.

After cleaning the data in the explore dataset GSE17901 by WGCNA, 5408 genes from 17 samples were analyzed for co-expression network construction. A scale-free network was constructed with a soft-threshold at nine, and a correlation coefficient threshold set at 0.85 (Figure 2A), and 15 related co-expression modules were obtained (Figure 2B). Four main clusters were observed. The turquoise module (1394 genes) was the biggest cluster, followed by the blue module (897 genes), brown module (793 genes), and yellow module (586 genes). All the ungrouped genes (199 genes) were included in the grey module.

### 3.2. Construction of Module-Trait Relationships and Detection of Key Modules

**Figure 3:**
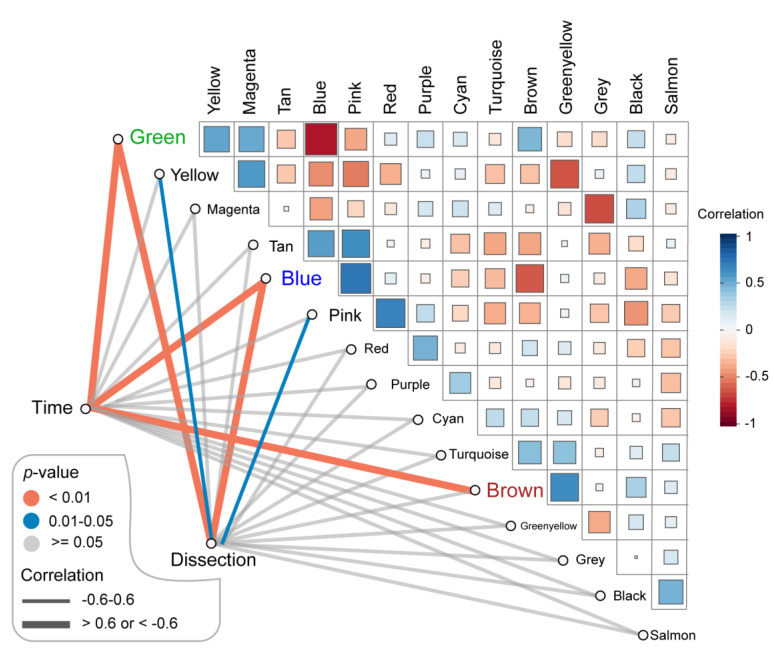
Identification of the key modules associated with AAA progression. Green, blue and brown modules were highly correlated (correlation > 0.6 or −0.6 and *p*-value < 0.01) to the time of sample collecting which stands for AAA progression. Besides, green and blue modules were also related to the dissection happening in the AAA sample (correlation > 0.6 or −0.6 and *p*-value < 0.01). AAA: abdominal aortic aneurysm.

The related sample traits (time—day 7, day 14, day 28; dissection of abdominal aorta) were obtained from the sample information in the GSE17901 dataset (Figure S1A). The relationships between these traits and each module were defined by the correlation between ME and sample traits (Figure 3, Figure S1B). These results indicated that three modules (blue, green, and brown) were strongly related to the time trait, representing the progression of AAA (Figure 3, Figure S2A–C). Blue and green modules also significantly correlated with the dissection sample trait (Figure 3, Figure S2D–E). Thus, the blue (897 genes), green (436 genes), and brown (793 genes) modules were defined as the key modules that were highly correlated with AAA.

### 3.3. Functional Enrichment Analysis of Genes in the Module

**Figure 4:**
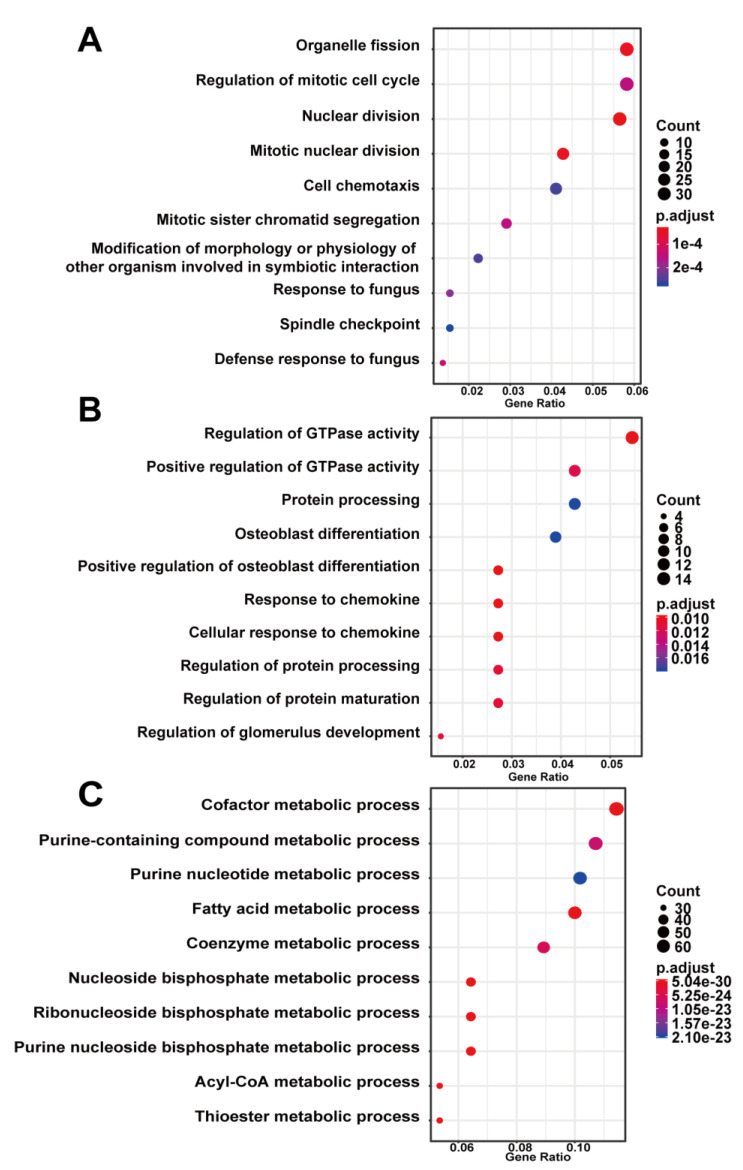
Gene ontology enrichment analysis of key modules of AAA progression. (**A**) blue module; (**B**) green module; (**C**) brown module. Count—the number of genes in the given GO term. Gene ration—the percentage of total genes in the given GO term.

**Figure 5:**
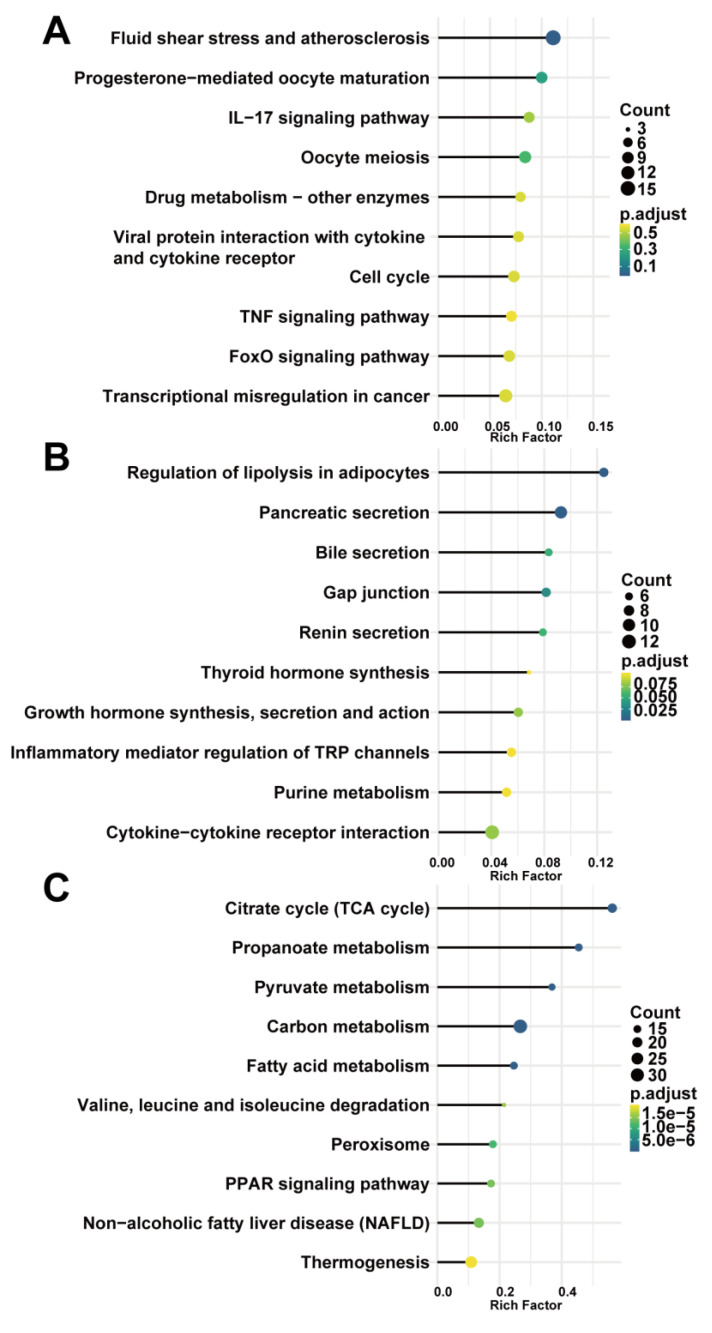
KEGG pathway enrichment analysis of key modules. (**A**) blue module; (**B**) green module; (**C**) brown module. Count—the number of genes in the given KEGG pathway. Rich factor—the ratio of the number of genes annotated in a pathway to the number of all genes annotated in this pathway.

To investigate the biological functions of key modules related to sample traits, we conducted GO and KEGG enrichment analysis for genes in every key module. The GO analysis showed that genes in the blue modules were mainly involved in the organelle fission, regulation of mitotic cell cycle, and nuclear division related to cell development or differentiation (Figure 4A). The green module was involved in GTPase activity (Figure 4B), and the brown module was clustered in cellular metabolic processes, especially cofactor metabolism, purine-containing compound metabolism, and purine nucleotide metabolism (Figure 4C). The results of the KEGG analysis revealed that the blue module was enriched in fluid shear stress and atherosclerosis pathway, highly related to the progression of AAA (Figure 5A). Genes in the green module were enriched in the regulation of lipolysis in the adipocyte pathway and the pancreatic secretion pathway (Figure 5B). The brown module was enriched in the citrate cycle (TCA cycle) pathway (Figure 5C).

### 3.4. Identification of Hub Genes in the Key Modules

To explore the hub genes that regulate AAA development, we imported the top 10% of genes with the highest connectivity into the String online database for protein–protein interaction detection, and networks were formed in Cytoscape (San Diego, CA, USA) (the PPI networks were stored in the NDEx: accessed on 11 December 2020 from https://bit.ly/37XZZWh; https://bit.ly/3a7Q2sc; https://bit.ly/38fyckz). With the cytoHubba plugin using the MCC method, the top 10 hub genes were identified in the key modules, namely, in the blue module (Ccr5, Fpr2, Ccr2, Fpr1, P2ry12, Hcar1, Ppbp, Aif1, Sirpb1b, Clec4n), green module (Gnai1, Adcy5, Adcy3, Rnase2a, Cxcl13, Clca1, Ear10, Ear1, Npr1, Ccl11), and brown module (Lpl, Dgat2, Fasn, Acacb, Lpin1, Acsl1, Mogat1, Lep, Ucp3, Pdk4) (Table 2).

### 3.5. Hub Genes Validation and Key Genes Selection

**Figure 6:**
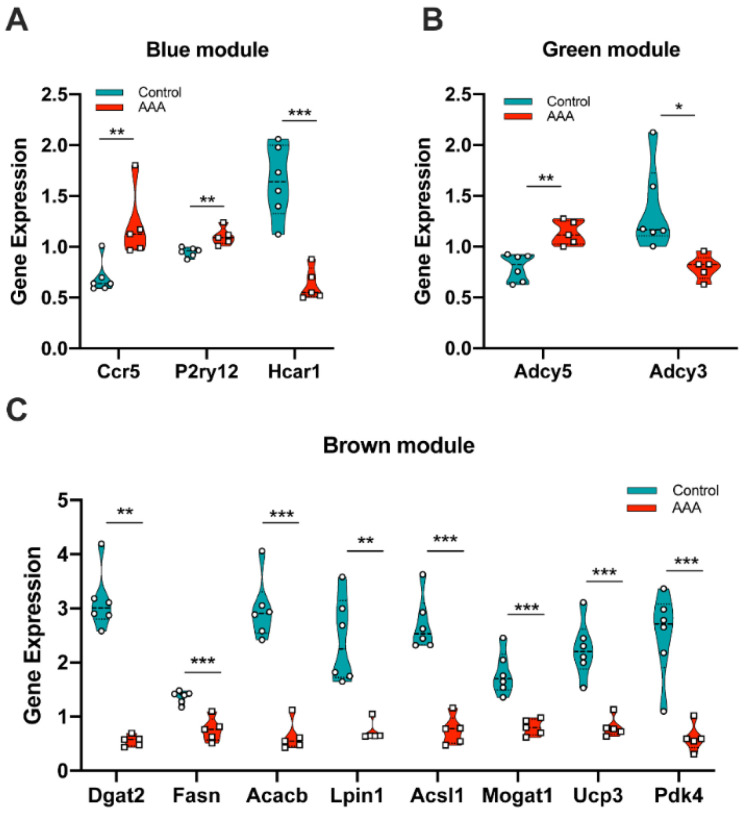
Validation of gene expression from hub genes in mouse dataset GSE12591. (**A**) Ccr5, P2ry12 and Hcar1 were differentially expressed in the blue module; (**B**) Adcy5 and Adcy3 were differentially expressed in the green module; (**C**) Dgat2, Fasn, Acacb, Lpin1, Acsl1, Mogat1, Ucp3 and Pdk4 were differentially expressed in the brown module. *: *p* < 0.05, **: *p* < 0.01, ***: *p* < 0.001 (Wilcoxon rank-sum test).

To further validate and evaluate the hub genes identified through the above analysis, the mouse dataset GSE12591 was checked using the same mouse angiotensin II-induced AAA model as GSE17901. In the blue module, Ccr5 and P2ry12 were significantly upregulated in the AAA group (Figure 6A), and Hcar1 was significantly down-regulated in the AAA group (Figure 6A). In the green module, Adcy5 and Adcy3 were the two significantly expressed genes (Figure 6B). All significantly expressed genes (Dgat2, Fasn, Acacb, Lpin1, Acsl1, Mogat1, Ucp3, Pdk4) in the brown module were down-regulated in the AAA group (Figure 6C). In the human AAA datasets GSE7084, GSE47472, and GSE57691, all of the significantly expressed genes were identified by comparing organ donors and AAA patients (Table 3). Considering the individual differences within each sample, genes expressed significantly in every human dataset were defined as human key genes. Finally, CCR5, ADCY5, ADCY3, ACACB, LPIN1, ACSL1, and UCP3 were the common genes that showed up both in the mouse AAA dataset and human AAA datasets and these were selected as the key genes in AAA progression.

### 3.6. Predication of Drug-Gene Interaction

**Figure 7:**
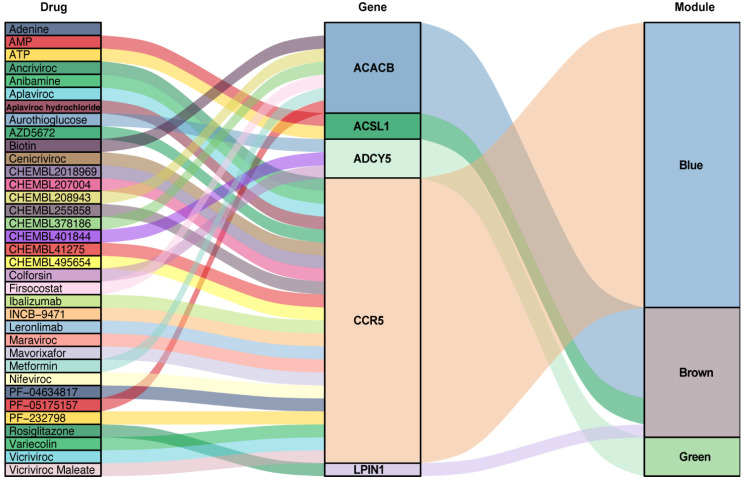
Drug–gene interaction prediction of key genes. Five key genes—ACACB, ACSL1, ADCY5, CCR5 and LPIN1 were targeted in the DGIdb database. A total of 35 potential target drugs/compounds were predicted from the database. AMP: Adenosine monophosphate; ATP: Adenosine triphosphate.

The seven key genes CCR5, ADCY5, ADCY3, ACACB, LPIN1, ACSL1, and UCP3 were used as the potential druggable targets for AAA treatment. The drug–gene interaction results from the DGIdb database revealed 35 potential target drugs/compounds for AAA treatment. Of these, 23 drugs targeted CCR5, among which maraviroc had the highest score of prediction; seven drugs targeted ACACB, two drugs each targeted ACSL1 and ADCY5, and one drug targeted LPIN1 (Figure 7, Table S1). No potential drugs could be identified for ADCY3 and UCP3

## 4. Discussion

In the present study, we used WGCNA analysis to identify the key genes involved in AAA progression and the drugs that target these genes, which could be potentially effective for the repression of AAA growth. WGCNA was performed on the available mouse dataset (GSE17901), where AAA samples were obtained at day 7, day 14, and day 28 from ApoE^−/−^ mice treated by angiotensin II or saline. We identified three modules (blue, green, and brown) as key modules that correlated closely with AAA growth. In these three modules, we further identified hub genes using Cytoscape software (San Diego, CA, USA) and validated the model in mouse and human datasets. Seven genes—CCR5, ADCY5, ADCY3, ACACB, LPIN1, ACSL1, and UCP3 were identified as the key genes in AAA progression. Finally, using the DGIdb database, we identified 35 drugs as potential candidates/compounds that could target the key genes and yield beneficial effects in treating AAA.

WGCNA is a systematic biological method that describes the gene co-expression pattern between different samples. It identifies gene sets with highly coordinated variations. The candidate biomarkers or targets of the disease are based on the connectivity between gene modules and sample traits. Compared to the traditional differential gene expression analyses, which focus solely on genes characterizing the difference between groups, WGCNA groups co-expressed genes in an unbiased manner into modules that can be connected to sample traits.

Among the 15 co-expression modules obtained by WGCNA, the blue, green, and brown modules were mostly related to the AAA progression. The enrichment analysis of these key modules’ biological functions and pathways revealed that genes in the blue module were mainly enriched in the cellular process, particularly the regulation of the mitotic cell cycle. This has also been reported in several studies. For instance, Butt et al. performed peripheral blood transcriptome profiling of individuals with AAA and healthy donors. They described that significantly expressed genes were enriched in this GO term [41]. Another study showed that the mitotic cell cycle was also significantly associated with dilated aortic perivascular adipose tissue [16]. The most enriched pathway of the blue module in KEGG was fluid shear stress and atherosclerosis. Several studies have shown the association of atherosclerosis disease with AAA [1,42]. Shear stress induced by abnormal blood flow was also previously reported to contribute to the growth or rupture of AAA [43,44]. The GO analysis of the green module showed that the biological process of GTPase activity was involved in AAA development. Dysregulation of GTPase activity would influence normal functions of endothelial cells and vascular smooth cells, including re-endothelialization, cell migration, and proliferation [45,46]. KEGG pathway enrichment of genes in the green module demonstrated that the regulation of lipolysis in the adipocyte pathway is also engaged in AAA growth. Adventitia of the aorta which contains the mass of adipocytes is a new direction of AAA research. One recent study revealed the key regulatory factors in perivascular adipose tissue of AAA [19]. Another study further proved that the increase in AAA diameter was correlated with lipid-related processes in the adventitia [18]. Results from functional enrichment analysis of the brown module indicated that some metabolic processes or pathways are also involved in AAA progression. In our study, cofactor metabolism was the most enriched process. This is in agreement with previously published studies that have shown that cofactors like cobalamin (vitamin B12) and glutathione could slow down the progression of AAA to some extent [47,48]. These findings confirm the involvement of the mitotic cell cycle, GTPase activity, and metabolic process in the pathogenesis of AAA.

The hub genes in the present study were selected by a combined analysis of gene intramodular connectivity and protein–protein interaction in the STRING database and Cytoscape software (San Diego, CA, USA). These selected hub genes were further confirmed in mouse and human datasets with gene differential expression analysis. Seven key genes were eventually identified—CCR5 from the blue module, ADCY5 and ADCY3 from the green module, ACACB, LPIN1, ACSL1, and UCP3 from the brown module. The vital role of CCR5, C-C motif chemokine receptor 5, in HIV-1 infection has been accepted since the discovery of this receptor [49]. It is expressed in many immune cells, including macrophages, T cells, and natural killer cells. CCR5 and its ligands regulate the inflammatory response by affecting the biological activities of the above-mentioned immune cells [50]. The results from GSE12591 identifying Ccr5 as a differential gene upregulated in the mouse aortas with aneurysms [25]. CCR5 signaling in the macrophage pathway was enriched by functional analyses of differential genes in GSE7084 [24]. Furthermore, patients with AAA frequently have CCR5 Delta 32 deletion mutations and are vulnerable to rupture of aneurysms [51]. Thus, CCR5 may be a potential biomarker for AAA progression and an indication of rupture. The hub gene ADCY5 (mouse—Adcy5) in the green module was related to mouse AAA progression and dissection. This was consistent with the findings by Phillips et al. which showed Adcy5 was one of the differentially expressed genes in the murine dissecting AAA [52]. ADCY3 is an enzyme that regulates the cyclic adenosine monophosphate (cAMP). Besides its role in AAA progression, loss of ADCY3 increases the risk of obesity and type 2 diabetes [53], and the single nucleotide polymorphisms of this gene are related to hypertension [54], which are the risk factors leading to the initiation of AAA [55]. LPIN1, ACSL1, and UCP3 were related to adipocyte differentiation and muscle growth [56,57,58,59,60], so dysregulation of these three genes may lead to AAA initiation, growth or rupture, as adipocytes residing in the perivascular tissue, and vascular smooth muscle cells play an important role in the development of AAA [19,60]. According to the reviewed literature, the remaining key gene ACACB had no apparent connection with AAA. This, however, requires further investigation to clarify its function in AAA progression.

So far, there is no effective drug therapy for the prevention of AAA progression or rupture. In this study, seven key genes were identified and used for predicting drug-gene interactions. A total of potential 35 drugs or compounds were presented in the DGIdb database. Most of these targeted the CCR5 gene. We checked these 35 candidates from the literature and ClinicalTrials.gov (accessed on 18 July 2020 from https://clinicaltrials.gov/), the largest clinical trials database containing over 329,000 trials worldwide. Five targetable drugs (PF-05175157, firsocostat, and metformin targeting ACACB; maraviroc targeting CCR5; rosiglitazone targeting LPIN1) were found to be used for AAA treatment. PF-05175157 and firsocostat are two novel acetyl-CoA carboxylase (ACC) inhibitors for lipid disorders [61,62], which could potentially rebalance dysregulated lipid metabolism in AAA to limit the development of the disease. Metformin is the first-line oral antidiabetic drug [63]. It also has proven effects on cardiovascular diseases through the reduction of inflammation and oxidative stress [64,65,66]. Several epidemiological studies have indicated that the use of metformin use could decrease yearly AAA growth [67,68]. Though maraviroc is a CCR5 antagonist prescribed for HIV-1 treatment, it could also be applied for AAA treatment since it was reported that maraviroc could reduce cardiovascular risk by modulation of atherosclerotic progression in vivo and in vitro [69,70]. Rosiglitazone (RGZ) is a potent peroxisome proliferator-activated receptor-γ (PPAR-γ) agonist that can protect against ischemia/reperfusion injury due to its anti-inflammatory effects [71]. It has been reported that RGZ reduces stent-induced neointimal formation by decreasing the inflammatory responses and vascular smooth muscle hyperplasia [72]. Through the same anti-inflammatory effect, RGZ could also inhibit the growth and rupture of mouse aortic aneurysms induced by angiotensin II and high cholesterol [73]. No drugs could be predicted for the ADCY3 and UCP3 genes. These two gene candidates will have to be evaluated as potential targets in AAA treatment in further studies.

Though our study is the first that performed WGCNA analysis with samples collected at different points of time in AAA growth, this study still has some limitations. Firstly, upon screening of the public database mouse dataset, GSE17901 was the only dataset available that allowed us to follow gene function over time and was used as an exploration dataset for WGCNA analysis. As a result, the sample size used for WGCNA analysis (*n* = 17) just passed the minimum official criteria (*n* = 15), therefore there may be noise for the biological network construction. The angiotensin II-induced AAA in mice may share similar features with human AAA, but the inherent pathology is different and thus, our results should be interpreted with caution. This study has indeed predicted interesting key genes involved in the progression of AAA and potentially useful drugs, however these findings should be validated further with in vitro and in vivo models of AAA.

In summary, this study identified key co-expression modules, key genes, and several critical biological processes related to AAA progression. With drug–gene interaction prediction, target drugs or compounds may provide the possibility of developing a medical treatment for AAA.

## 5. Conclusions

Our study using WGCNA analyses revealed seven key genes (CCR5, ADCY5, ADCY3, ACACB, LPIN1, ACSL1, UCP3) in three modules correlated to AAA progression. Mitotic cell cycle, GTPase activity, and metabolic process were involved in the pathogenesis of AAA. The therapeutic potential of several predicted drugs for the treatment of AAA could be further explored.

## Figures and Tables

**Table 1:** GSE datasets included in the study.

Catalog.	GSE Dataset	Organism	Sample Number *	PMID
Explore dataset	GSE17901	Mouse	AAA day7: 7, AAA day14: 5, AAA day28: 6	21712436
Validate dataset	GSE12591	Mouse	Control: 6, AAA: 5	19580648
	GSE7084	Human	Donor: 10, AAA: 9	17634102
	GSE47472	Human	Donor: 8, AAA: 14	NA
	GSE57691	Human	Donor: 10, AAA: 49	NA

*: Number of samples (control or AAA) used in this study; NA: not applicable.

**Table 2:** Top 10 ranked genes in key modules with the MCC method in cytoHubba.

Catalog	Key Modules		
	Blue	Green	Brown
Top 10 Gene	Ccr5	Gnai1	Lpl
	Fpr2	Adcy5	Dgat2
	Ccr2	Adcy3	Fasn
	Fpr1	Rnase2a	Acacb
	P2ry12	Cxcl13	Lpin1
	Hcar1	Clca1	Acsl1
	Ppbp	Ear10	Mogat1
	Aif1	Ear1	Lep
	Sirpb1b	Npr1	Ucp3
	Clec4n	Ccl11	Pdk4

**Table 3:** Significantly expressed hub genes in human AAA datasets.

Datasets	Key Modules		
	Blue	Green	Brown
GSE7084	CCR5, CCR2, FPR2, FPR1, AIF1	GNAI1, RNASE2, NPR1	NA
GSE47472	CCR2, FPR2, PPBP	GNAI1, RNASE2, CLCA1, LYVE1	LPIN1, UCP3
GSE57691	CCR2, FPR2, PPBP, CLEC6A, SIRPB1	ADCY5, ADCY3, CXCL13, CLCA1, CCL11	ACACB, LPIN1, ACSL1, LEP
Human	CCR5, CCR2, FPR2, PPBP, AIF1, CLEC6A, SIRPB1, FPR1	GNAI1, RNASE2, NPR1, CLCA1, LYVE1, ADCY5, ADCY3, CXCL13, CCL11	ACACB, LPIN1, ACSL1, LEP, UCP3

## Data Availability

All datasets of this study are available in the GEO database (https://www.ncbi.nlm.nih.gov/geo/, accessed on 13 May 2021).

## Supplementary Materials

The following are available online at https://www.mdpi.com/article/10.3390/biomedicines9050546/s1, Figure S1: Sample clustering and module relations to sample traits. (A) Sample dendrogram and trait heatmap. The color intensity of time was proportional to the day of the sample collected. The red color in dissected represents the occurrence of dissection in the sample; (B) Module trait relationships. Each row corresponds to a module eigengene (ME) and each column to a sample trait, Figure S2: Correlation of the module membership and the gene significance. (A–C) The relationship between gene significance of time and module membership; (D–E) The relationship between gene significance of dissection and module membership. The color indicates the module, and the dot indicates the gene within the module., Table S1: Potential target agents identified based on drug-gene interaction in DGIdb database.
