# Supplementary material for: Weighted Gene Co-Expression Network Analysis Reveals Key Genes and Potential Drugs in Abdominal Aortic Aneurysm
Source: Biomedicines. 2021 May 13;9(5):546. doi: 10.3390/biomedicines9050546 (PMC8152975; doi:10.3390/biomedicines9050546)
Supplement: Supplementary file 1 [file biomedicines-09-00546-s001.zip › biomedicines-1199468-supplementary 2.pdf]

Supplementary Figures

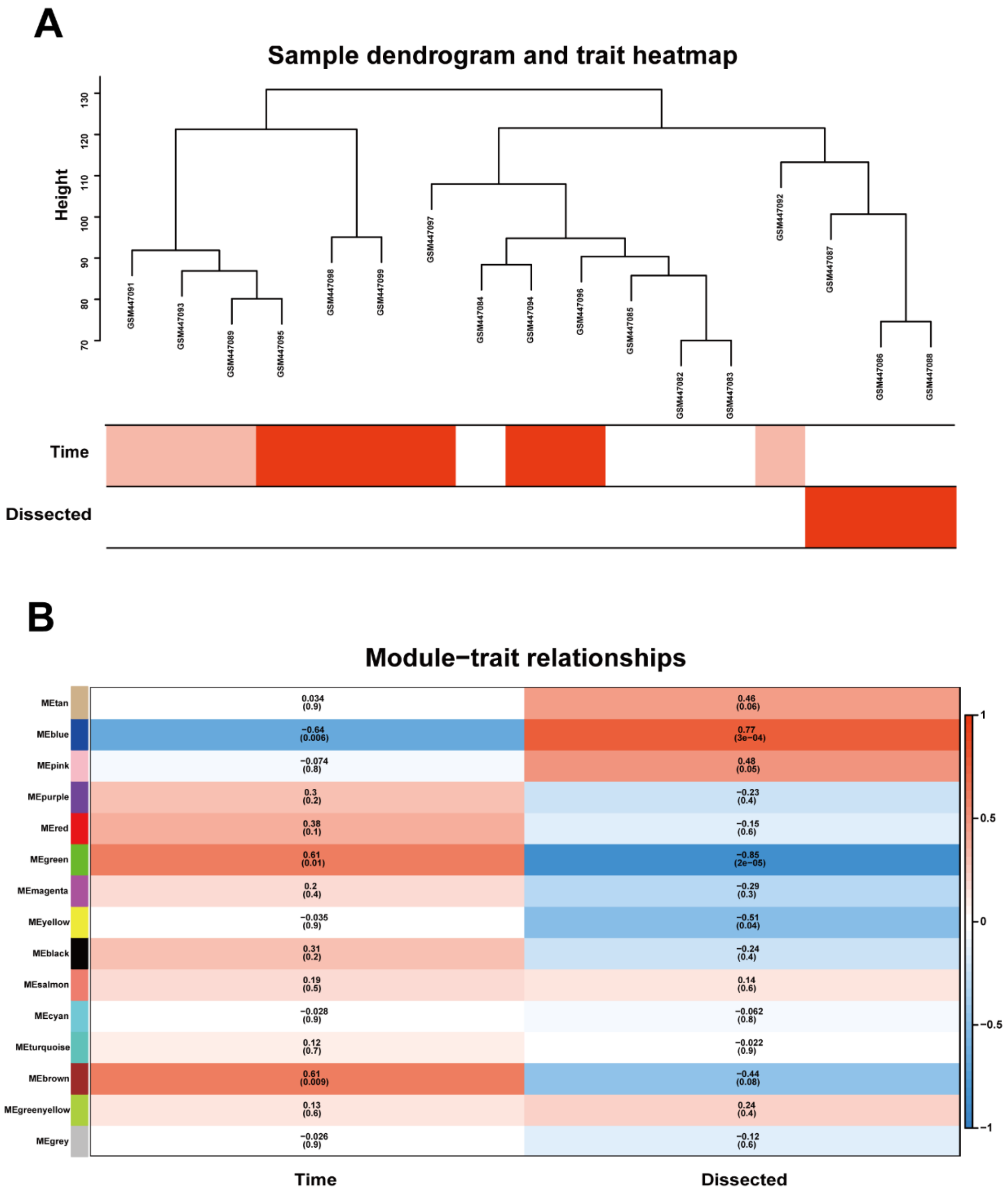

Supplementary Figure S1. Sample clustering and module relations to sample traits. (A) Sample dendrogram and trait heatmap. The color intensity of time was proportional to the day of the sample collected. The red color in dissected represents the occurrence of dissection in the sample; (B) Module trait relationships. Each row corresponds to a module eigengene (ME) and each column to a sample trait.

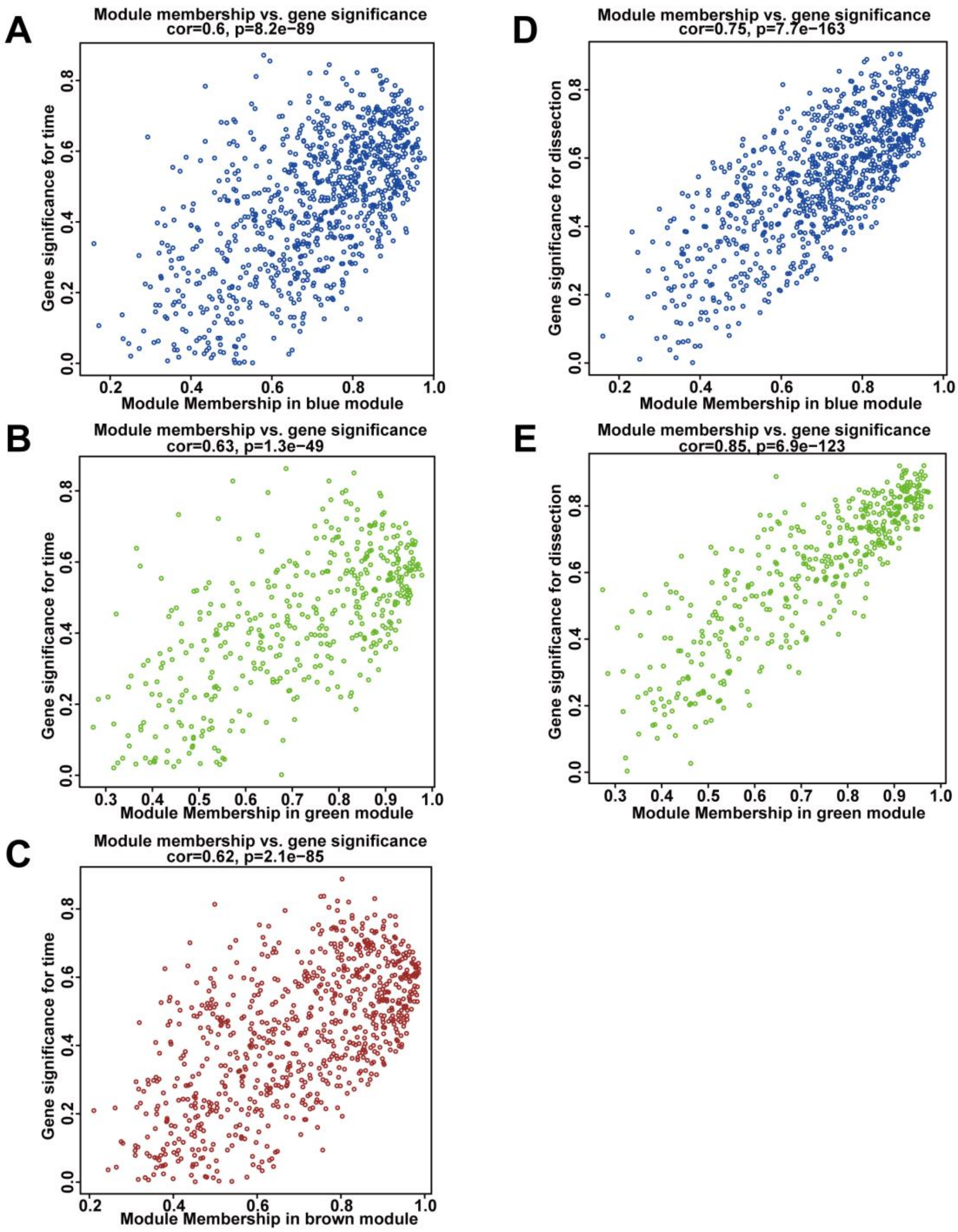

Supplementary Figure S2. Correlation of the module membership and the gene significance. (A-C) The relationship between gene significance of time and module membership; (D-E) The relationship between gene significance of dissection and module membership. The color indicates the module, and the dot indicates the gene within the module.

Supplemenary Table

Table S1. Potential target agents identified based on drug-gene interaction in DGIdb database.

Gene symbol	Gene name	Target agent	Drug-gene Interaction	Query Score	Interaction Score
ACACB	Acetyl-CoA Carboxylase Beta	CHEMBL378186	NA	4.45	8.74
		Adenine	NA	4.45	8.74
		PF-05175157	NA	4.45	8.74
		CHEMBL208943	NA	4.45	8.74
		Firsocostat	Allosteric modulator	2.22	4.37
		Biotin	Cofactor	1.33	2.62
		Metformin	NA	0.11	0.22
ACSL1	Acyl-CoA Synthetase Long Chain Family Member 1	Adenosine monophosphate	NA	0.89	6.12
		Adenosine triphosphate	NA	0.23	1.61
ADCY5	Adenylate Cyclase 5	CHEMBL401844	NA	2.22	10.2
		Aurothioglucose	NA	1.48	6.8
		Colforsin	NA	0.74	3.4
CCR5	C-C motif chemokine receptor 5	Maraviroc	Antagonist	31.15	19.47
		Vicriviroc	Antagonist	26.7	16.69
		Leronlimab	Antagonist	22.25	13.91
		CHEMBL207004	Antagonist	17.8	11.13
		INCB-9471	Antagonist	13.35	8.34
		Cenicriviroc	Antagonist	8.9	5.56
		AZD5672	Antagonist	8.9	5.56
		Aplaviroc	Antagonist	8.9	5.56
		Ancriviroc	Antagonist	4.45	2.78
		Vicriviroc Maleate	Antagonist	4.45	2.78
		Anibamine	NA	4.45	2.78
		Variecolin	NA	4.45	2.78
		CHEMBL255858	NA	4.45	2.78
		CHEMBL495654	NA	4.45	2.78
		PF-232798	NA	4.45	2.78
		Nifeviroc	NA	4.45	2.78
		Aplaviroc hydrochloride	Antagonist	2.22	1.39
		CHEMBL41275	Antagonist	2.22	1.39
		PF-04634817	Antagonist	2.22	1.39
		CHEMBL2018969	Antagonist	2.22	1.39
		Mavorixafor	NA	2.22	1.39
		Ibalizumab	Antagonist	1.11	0.7
LPIN1	Lipin 1	Rosiglitazone	NA	0.28	3.82
